# Sepsis Resuscitation: Time to Embrace a Restrictive Fluid Strategy?

**DOI:** 10.1016/j.acepjo.2024.100040

**Published:** 2025-01-21

**Authors:** Hezael Agustín Toledo-Palacios, Orlando Rubén Pérez-Nieto, Rafael Reyes-Monge, Ignacio Rodríguez-Guevara, Nicholas M. Mark

**Affiliations:** 1 Department of Emergency Medicine, Hospital General de Subzona IMSS No. 9, Sonora, México; 2 Intensive Care Unit, Hospital General San Juan del Río, Querétaro, México; 3 Division of Critical Care Medicine, Department of Medicine, Swedish Medical Center, Seattle, Washington, USA

Dear Editor,

We read with great interest the article by Jayaprakash et al^1^ on state-of-the-art sepsis care for emergency physicians and would like to comment on intravenous fluid therapy management.

The authors advocate administering 30 mL/kg of crystalloids for sepsis-induced hypotension and hypoperfusion, which is in line with the Surviving Sepsis Campaign guidelines.^2^ However, this recommendation has been downgraded from moderate to weak in recent updates, reflecting a lack of robust evidence supporting its benefits. Recent trials and meta-analyses challenge the routine use of liberal fluid therapy.

The Conservative versus Liberal Approach to Fluid Therapy of Septic Shock in Intensive Care trial^3^ compared restrictive versus liberal fluid therapy, but differences in fluid volumes were minimal, with both groups receiving substantial prerandomization fluids (>2 L), limiting meaningful comparisons. Similarly, the Crystalloid Liberal Or Vasopressors Early Resuscitation in Sepsis trial^4^ failed to demonstrate superiority of liberal therapy over restrictive fluid therapy. A systematic review^5^ of 11 randomized controlled trials (RCTs) involving 4121 patients found no significant differences between liberal and restrictive strategies in 30-day mortality (odds ratio, 0.73; 95% CI, 0.30-1.80; *P* = .50), adverse events, or hospital length of stay. Notably, restrictive approaches showed trends toward better outcomes, including reduced need for mechanical ventilation and fewer adverse events.

Given these findings, fluid therapy should be judiciously administered, tailoring resuscitation to individual patient needs. Restrictive fluid strategies could be particularly beneficial in patients with a pulse pressure <30 mm Hg and positive response parameters such as plethysmographic variability index,^6^ a noninvasive tool suitable for emergency settings. Passive leg raising combined with capillary refill time assessment^7^ may further guide fluid responsiveness despite known limitations.

Current US shortages of intravenous fluids underscore the need for strategic use. Disruptions caused by natural disasters, such as Hurricane Helene, have severely impacted production, exacerbating supply challenges amid rising seasonal demand. This practical constraint aligns with clinical evidence favoring restrictive fluid administration.

Moreover, early norepinephrine initiation^8^ may enhance outcomes by reducing total fluid volumes and improving organ perfusion. Data indicate lower volumes of fluid administration at vasopressor initiation (median [range]: 0 [0-510] vs 1500 [650-2300] mL; *P* < .001) and during the first 8 hours (median [range]: 1100 [500-1900] vs 2600 [1600-3800] mL; *P* < .001). Early norepinephrine has also been associated with reduced acute pulmonary edema (odds ratio, 0.43; 95% CI, 0.25-0.74) and increased ventilator-free and vasopressor-free days in a meta-analysis of RCTs.^9^ A bolus of 4 mL/kg of crystalloid fluid appears to be effective and reduces the total fluid volume administered.^10^

In the Figure, we present a comparison between the traditional approach to fluid therapy in sepsis and the rational approach, highlighting key differences in strategy and clinical decision-making.

**Figure:**
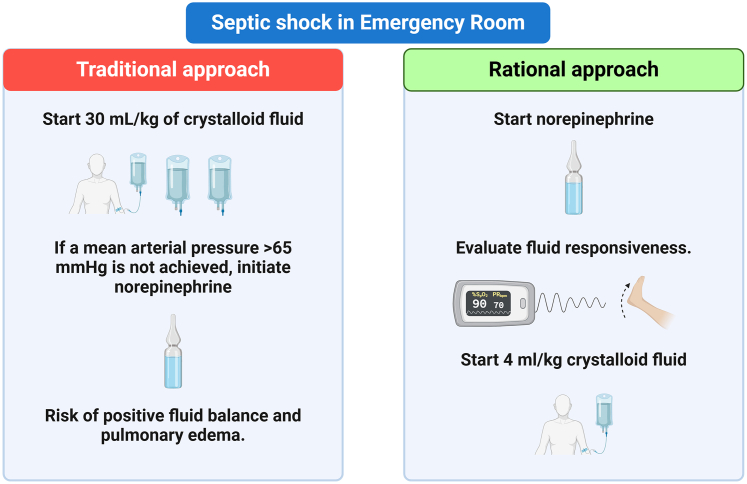
Traditional vs rational fluid management in sepsis: a comparative approach (created in BioRender.com).

In conclusion, sepsis management in the emergency department should emphasize rational fluid use, guided by reliable parameters such as pulse pressure and plethysmographic waveform analysis. Early norepinephrine administration may further optimize outcomes, reducing unnecessary fluid use and associated complications. This strategic approach improves clinical efficacy and resource stewardship, ultimately benefiting patients and health care systems.

## Funding and Support

This research did not receive any specific grant from funding agencies in the public, commercial, or not-for-profit sectors.

## Conflict of Interest

All authors have affirmed they have no conflicts of interest to declare.
